# The effectiveness of immunomodulatory therapies for patients with repeated implantation failure: a systematic review and network meta-analysis

**DOI:** 10.1038/s41598-022-21014-9

**Published:** 2022-11-01

**Authors:** Mengqi Liu, Yuan Yuan, Yan Qiao, Yuzhu Tang, Xi Sui, Ping Yin, Dongzi Yang

**Affiliations:** 1 Kapok Zhucheng Medical Clinic, No. 302, No. 9, Huaqiang Road, Tianhe District, Guangzhou, 510623 China; 2 Shanghai Kapok Integrated Traditional Chinese and Western Medicine Clinic Co., Ltd., 3rd Floor, No. 21 Pudong South Road, Pudong New Area, Shanghai, 200126 China; 3 Shenzhen Kapok Health Medical Co., Ltd. Kapok Clinic, L2-12, Shenye Tairan Building, Tairan 8th Road, Shatou Street, Futian District, Shenzhen, 518042 China; 4 Guangzhou Kapok Medical Investment Co., Ltd., Room 116 and 117, No. 173, South 2nd Road, Yuncheng, Baiyun District, Guangzhou, 510405 China; 5 Department of Obstetrics and Gynecology, Sun Yat-Sen Memorial Hospital, Sun Yat-Sen University, 107 Yanjiang West Road, Guangzhou City, 528406 Guangdong Province China

**Keywords:** Immunotherapy, Drug screening

## Abstract

This meta-analysis analyzed the clinical pregnancy outcomes of repeated implantation failure (RIF) patients treated with immunomodulatory therapies. Publications (published by August 16, 2021) were identified by searching the PubMed, Embase, and Web of Science databases. The quality of the studies was evaluated with the Cochrane bias risk assessment tool, and a network meta-analysis was performed with Stata 14.0. The outcomes were clinical pregnancy rate (CPR), live birth rate (LBR), and implantation rate (IR). The results of our network meta-analysis of 16 RCTs (including 2,008 participants) show that PBMCs, PRP, and SC-GCSF can significantly improve the CPR compared with LMWH (PBMCs: OR 2.15; 95% CI 1.21–3.83; PRP: OR 2.38; 95% CI 1.08–5.24; SC-GCSF: OR 2.46; 95% CI 1.05–5.72). The LBR of PRP was significantly higher than those of IU-GCSF (OR 3.81; 95% CI 1.22–11.86), LMWH (OR 4.38; 95% CI 1.50–12.90), and intralipid (OR 3.85; 95% CI 1.03–14.29), and the LBR of PBMCs was also significantly better than that of LMWH (OR 2.35; 95% CI 1.14–4.85). Furthermore, PRP treatment significantly improved the IR compared with LMWH treatment (OR 2.81; 95% CI 1.07–7.4). The limited evidence from existing RCTs suggests that PBMCs and PRP are the best therapeutic options for RIF patients. However, owing to the quantity limitation, more top-quality research is required to obtain additional high-level evidence.

## Introduction

Repeated implantation failure (RIF) is the inability to achieve a clinical pregnancy after multiple cycles of in vitro fertilization and the cumulative transfer of multiple high-quality embryos in patients using assisted reproductive techniques^1^. Different academic organizations and researchers have attempted to propose clear diagnostic criteria; however, because of the complexity of the causes of RIF and the high diversity of affected patients, no consensus has been generated to date. The current widely used definition of RIF, proposed by Coughlan et al.^2,3^, is a lack of successful clinical pregnancy in a woman under the age of 40 years after the transfer of at least four good-quality embryos over a minimum of three fresh or frozen cycles. Implantation is a very complicated process, and there are numerous factors, of either maternal or embryonic origin, that contribute to RIF. The embryo, as a homozygous hemizygous antigen, is subject to a variety of factors for its successful implantation^1,4^. After an embryo is transferred into the uterine cavity, the endometrium must be acceptable for embryo synchronization, and the maternal immune system must tolerate the continued presence of the paternal alloantigen during the pregnancy^5^. Many potential factors, such as uterine abnormalities, hormonal or metabolic disorders, infections, immunological factors, thrombophilias, severe male factors, or an abnormal immunological response, can contribute to defective maternal–fetal immunotolerance and impaired endometrium receptivity.

There are a variety of immune cells in the endometrium, including natural killer (NK) cells, macrophages (Mφ), dendritic cells (DCs), and T cells, all of which play a role in regulating endometrial receptivity and embryo implantation^6^. In addition, immune-related cytokines in the intima, including interleukin (IL)-6, IL-10, IL-15, IL-17, tumor necrosis factor alpha (TNF-α), interferon gamma (IFN-γ), and nuclear factor kappa B (NF-κB), are also involved in determining the success of embryo implantation and development^7,8^. In order to restore the underlying immunological imbalance, some immunomodulatory therapies have been introduced to enhance clinical outcomes in women with unexplained RIF^9–11^. These immunomodulatory therapies include low-molecular-weight heparin (LMWH), intravenous immunoglobulin (IVIG), intrauterine (IU) human chorionic gonadotropin (hCG), subcutaneous (SC) or IU infusion of granulocyte colony-stimulating factor (GCSF), peripheral blood mononuclear cells (PBMCs), and intrauterine autologous platelet-rich plasma (PRP)^12–15^. However, there is conflicting evidence supporting the efficacy of these treatments, and the comparable efficacy of these immunomodulatory therapies in the rescue of RIF has not been determined.

Therefore, our network meta-analysis study compared the efficacy of the most widely used immunomodulatory therapies for RIF treatment to provide an evidence basis for theclinical application.

## Methods

This study was conducted in accordance with the Preferred Reporting Items for Systematic Reviews and Meta-analyses (PRISMA) reporting guidelines (Supplementary Material 1).

### Search strategy

**Figure 1:**
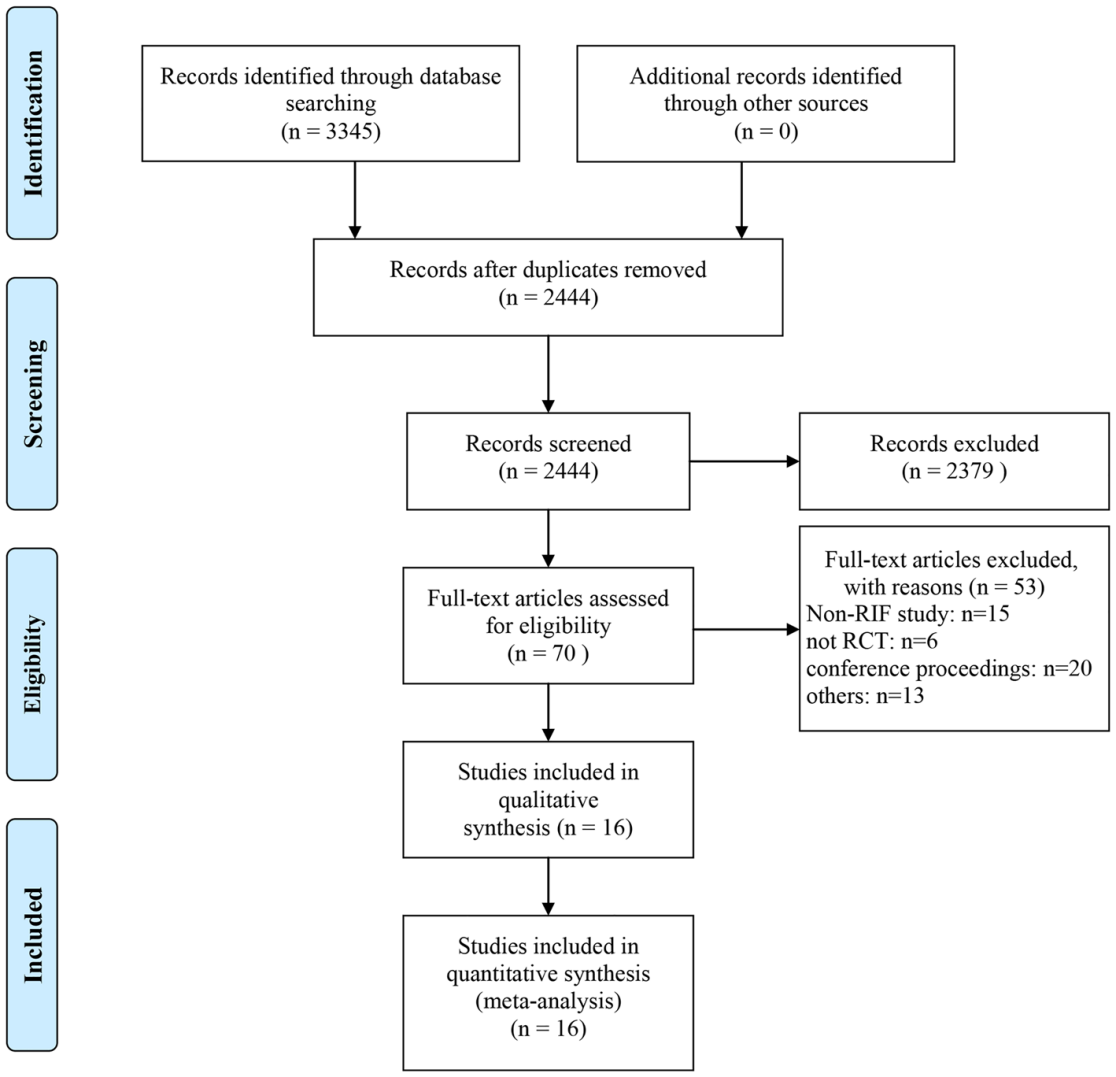
The flow diagram of the selection process for this study.

Publications were identified for inclusion in this meta-analysis by searching the PubMed, Embase, and Web of Science (all databases) databases (see screening flow chart in Fig. 1). The last search date was August 16, 2021, and the search language was limited to English. The following terms were applied for this search: “repeated implantation failure,” “recurrent implantation failure,” “intravenous immunoglobulin,” “PBMC,” “G-CSF,” “IVIG,” “PRP,” “intralipid,” “glucocorticoid,” “hCG,” “LMWH,” and “aspirin” (See detailed retrieval strategies in Supplementary Material 2).

### Selection criteria

Two authors independently screened the literature compiled in EndNote software. Any disagreements between the two authors regarding the inclusion of a publication were resolved by discussion with the third author to reach a consensus. Strict literature inclusion and exclusion criteria were established. The selected publications were required to meet the following criteria: (1) The study was a randomized controlled trial in which the experimental group was treated with an immunomodulatory therapy and the control group was given the standard care/placebo/no immunomodulatory therapy; (2) The study participants had two or more episodes of implantation failure; and (3) The study included at least one of three defined outcome metrics (clinical pregnancy rate [CPR], live birth rate [LBR], and implantation rate [IR]).

Research was excluded if it met any of the following conditions: (1) the data were incomplete or unable to be used for statistical analysis; or (2) the publication was a non-authoritative document, such as a review, letter, conference abstract, or review.

### Data extraction

Two authors independently derived the relevant data from the qualified literature. The extracted content included: first author, publication year, research type, total number of included participants, mean participant age, RIF inclusion criteria, and outcome indicators. The Cochrane bias risk assessment tool was utilized to access the quality of the identified randomized control trials. If the opinions of the two authors differed, the third author would make a judgment.

### Statistical analysis

Stata 14.0 was used to conduct the network meta-analysis under the consistency model. The odds ratio (OR) and 95% confidence interval (CI) were calculated for dichotomous outcomes. A paired meta-analysis was performed using a fixed-effects model based on the main results. I^2^ was used to assess the heterogeneity, and I^2^ ≥ 50% was taken to indicate statistical heterogeneity. When there was no closed triangle or quadratic loop connecting the three arms, the inconsistency between direct and indirect comparisons was assessed using a node-splitting method. The surface under the cumulative ranking (SUCRA) was used to evaluate the likelihood that each intervention was the most beneficial or safest treatment. A greater SUCRA value was taken to indicate a higher treatment efficacy. A comparison-correction funnel chart was used to assess the publication bias. *P* > 0.05 was taken to indicate no statistical inconsistency.

## Results

### Baseline characteristics of the included studies

Overall, 3,350 documents were identified by applying our search criteria. Of these, 901 duplicate articles were eliminated, and 2,379 publications were eliminated after examining the title and abstract. After reading the full text of the remaining 70 publications, 16 studies that met our requirements were finally included in this meta-analysis (Fig. 1, Supplementary Material 3). Among these 16 studies, which included 2,008 participants^16–31^, three examined LMWH, six investigated GCSF, four trialed PBMCs, one tested hCG, one studied intralipid, and two assessed PRP. Because the identified studies examining glucocorticoid and IVIG did not meet our selection criteria, so no studies on these therapies were included in our meta-analysis. The mean age of the study population ranged from 30.51 to 37.8.

**Figure 2:**
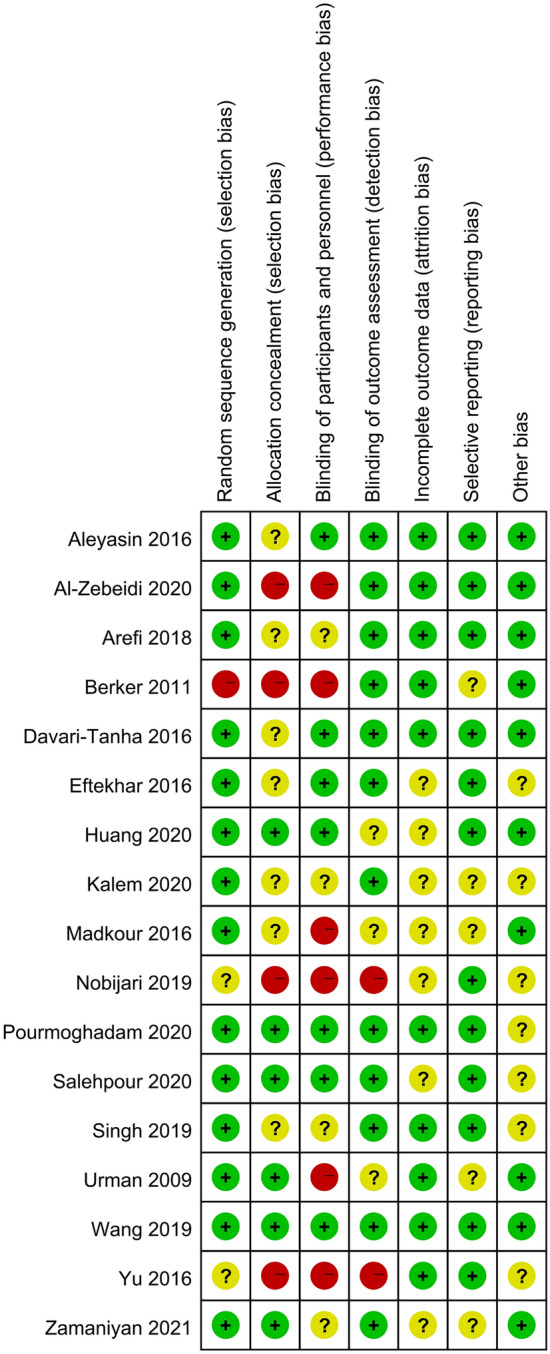
The risk of bias summary, review authors´ judgements about each risk of bias item for every included study.

**Figure 3:**
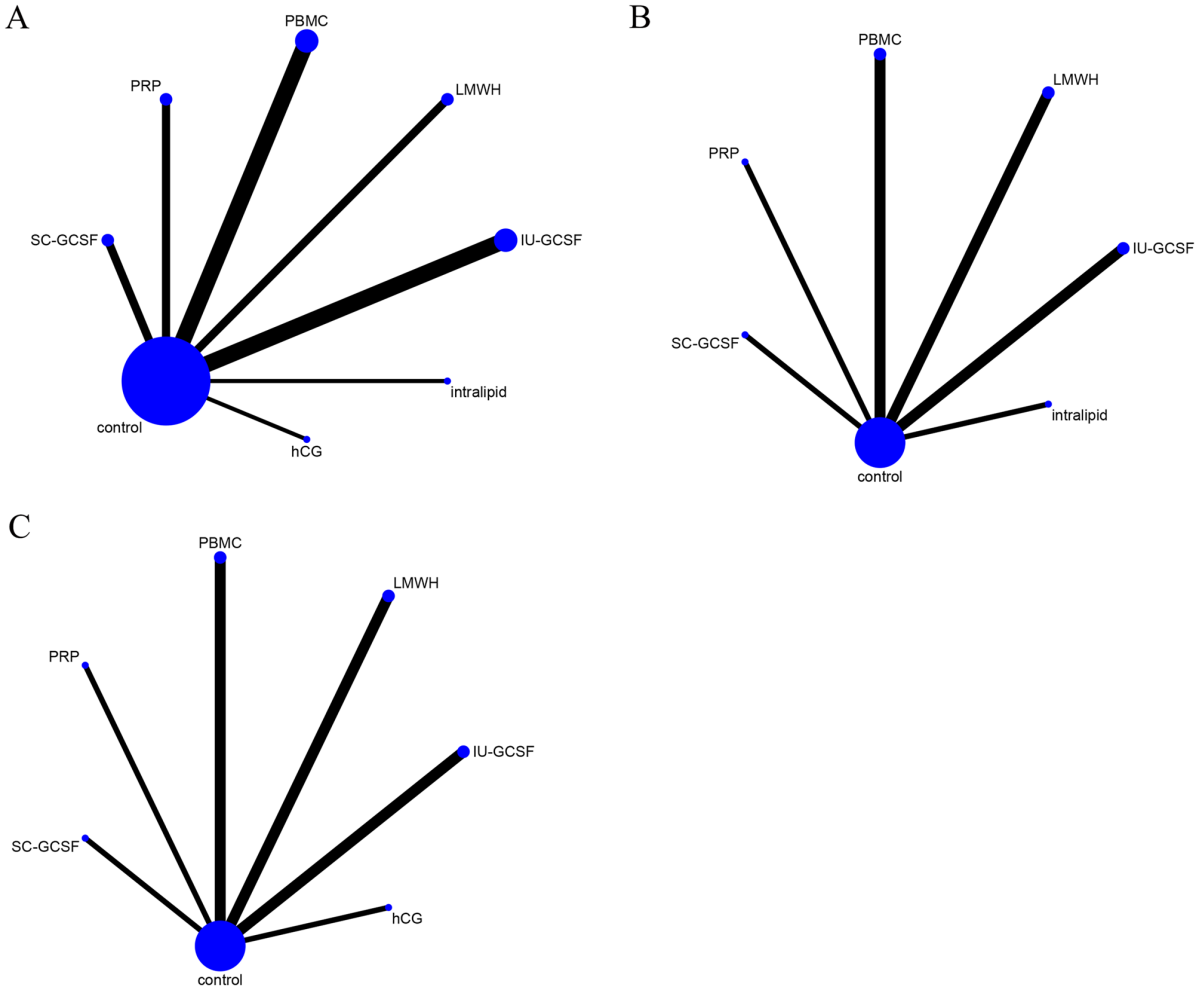
Evidence network diagram of the network meta-analysis comparisons.

The baseline characteristics of the involved studies were presented in Table 1. An assessment of the quality of these selected studies, as determined using the Cochrane risk of bias tool, was presented in Fig. 2. The network of eligible comparisons for each outcome was shown in Fig. 3. There was no closed loop between interventions, which suggested that all of these pairwise comparisons were indirect. Therefore, the statistical analysis was performed directly under the consistency model.

**Table 1:** Baseline characteristics of included studies.

Study	Study design	RIF criteria	Interventions	No. of patients	Age (Year)	BMI (kg/m^2^)	No. of transferred embryos (mean)
Urman 2009	Randomized open-labeled pilot trial	Three or more previously failed fresh embryo transfer cycles	LMWH (administered LMWH at a dose of 1 mg/kg/day starting on the day after oocyte retrieval)	75	34.0 ± 5.0	–	2.6 ± 0.7
			Control (received no medication besides progesterone gel on the day after oocyte retrieval)	75	34.8 ± 5.8	–	2.6 ± 0.8
Berker 2011	Prospective, quasi-randomized, controlled study	at least two consecutive failed cycles of intracytoplasmic sperm injection and embryo transfer (ICSI-ET)	LMWH (administered LMWH at a standard dose of 40 mg/0.4 mL per day starting on the day of oocyte retrieval)	104	31.3 ± 4.9	–	2.4 ± 0.6
			Control (No LMWH treatment)	103	31.2 ± 5.0	–	2.5 ± 0.6
Aleyasin 2016	Prospective randomized openlabel controlled trial	Failure of implantation in at least three consecutive IVF attempts, in which three embryos of high-grade quality are transferred in each cycle	subcutaneous GCSF (A single dose of 300 μg G-CSF administered subcutaneously 1 h before the embryo transfer)	56	33.5 ± 4.2	–	2.3 ± 0.6
			Control (did not receive any additional treatment before the embryo transfer)	56	32.4 ± 5.2	–	2.5 ± 0.6
Davari-Tanha 2016	Randomized double blind placebo control trial	three times implantation failure when there was history of transferring at least four good quality embryos without uterine or thrombophilic factors	intrauterine GCSF (At the time of oocyte retrieval one ml of G-CSF (300 μg/ml) was administered by a Trans cervical Cook catheter for embryo transfer slowly into uterine cavity)	40	35.5 ± 4.32	25.2 ± 1.8	–
			Control (a catheter pass through the cervix without any injection)	20	35.4 ± 4.01	24.8 ± 1.3	–
Eftekhar 2016	Randomised controlled trial	two or more episodes of implantation failure	intrauterine GCSF (received uterine infusion of 300 μg (0.5 ml) recombinant human GCSF (300 μg) by the use of IUI catheter after ovarian puncture under general anesthesia)	45	32.55 ± 4.61	–	2.11 ± 0.77
			Control (the standard treatment)	45	31.75 ± 5.16	–	2.35 ± 0.71
Madkour 2016	Randomised controlled trial	at least two previous failures of implantation after IVF/intra-cytoplasmic spermatozoa injection (ICSI) (mean = 3)	PBMC (Intrauterine administration of PBMC prior to fresh embryo transfer)	27	34.74 ± 4.17	–	–
			Control (no treatment group without receiving any cell transfer prior to embryo transfer)	27	34.44 ± 3.86	–	–
Yu 2016	Prospective randomized study	Patients who had not experienced successful pregnancy despite three or more IVF-ET sessions	PBMC (intrauterine administration of autologous PBMC activated by HCG in vitro before ET)	93	31.08 ± 3.95	–	–
			Control (undergoing ET without a previous intrauterine administration of autologous PBMC)	105	31.22 ± 5.12	–	–
Arefi 2018	Randomised controlled trial	the history of more than two previous IVF/Intracytoplasmic sperm injection-embryo transfer (ET) failures despite transfer of at least two good-quality embryos in each attempt	subcutaneous GCSF (receive 300 μg (0.5 ml) recombinant human G-CSF subcutaneously which was injected 30 min before blastocyst embryo transfer)	32	34.53 ± 5.50	–	3.31 ± 0.85
			Control (routine procedure)	20	34.05 ± 6.5	–	3.20 ± 0.95
Nobijari 2019	Prospective randomized study	a history of at least one RIF	PBMC (a blood sample was collected 5 days before the scheduled frozenthawed embryo transfer; PBMCs were isolated using Ficoll separation and then cultured for 72 h. Two days prior to embryo transfer, 0.4 ml of cultured PBMCs were transferred into the patient’s uterus)	122	35.21 ± 4.84	–	–
			Control (no treatment)	128	34.55 ± 5.03	–	–
Wang 2019	Prospective randomized-controlled trial	Failure of implantation in at least 4 consecutive IVF attempts, in which 1 embryos of high-grade quality are transferred in each cycle	hCG (The hCG + G2 fluid was prepared on the day of embryo transfer, and 40 μL of which was injected at an intrauterine site at 3 min before embryo transfer)	69	31.35 ± 3.18	22.3 ± 3.25	–
			Control (the G2 fluid was prepared on the day of embryo transfer, and 40 μL of which was injected at an intrauterine site at 3 min before embryo transfer)	68	31.7 ± 3.56	22.7 ± 3.61	–
Al-Zebeidi 2020	Randomised controlled trial	a history of three or more RIF undergoing ICSI cycles	Intralipid (received intralipid 20% 100 ml diluted in 500 ml normal saline for infusion therapy on the day of embryo transfer (ET) and repeated dose was administered on the day of the pregnancy test)	71	35.32 ± 4.23	28.30 ± 4.66	–
			Control (underwent the standard ICSI cycle without intralipid infusion therapy)	71	35.21 ± 4.77	28.30 ± 4.66	–
Huang 2020	Prospective randomized single-blind study	more than two failed implantations (each time containing at least one high-quality embryo	intrauterine GCSF (administered a 1-ml uterine infusion of recombinant human G-CSF (150 mg, 1 ml) through an intrauterine insemination catheter.)	52	32.09 ± 4.21	21.24 ± 2.29	
			Control (an intrauterine infusion of physiological saline before embryo transfer)	52	32.07 ± 4.36	21.51 ± 2.90	
Kalem 2020	Prospective randomized controlled trial	the failure to achieve a clinical pregnancy after the transfer of at least four good-quality embryos in a minimum of three fresh or frozen cycles to a woman under the age of 40 years	intrauterine GCSF (received G-CSF once a day on hCG day, before hCG injection. The procedure involved the administration of 30 mIU of Leucostim (G-CSF 30mIU/mL) through slow infusion into the endometrial cavity using a soft embryo transfer catheter)	82	34.61 ± 4.77	25.92 ± 4.44	–
			Control (normal saline of 1 mL was infused into the endometrial cavity of patients in the same way as the study group)	75	34.92 ± 5.60	24.94 ± 4.92	–
Pourmoghadam 2020	Double-blind randomized control trial	at least three previous failures of IVF/ET therapy	PBMC (PBMCs (15–20 × 106 cells) were suspended in 500 μl PBS and was gently administered to the uterine cavity two days before ET using an embryo transfer catheter)	50	33.42 ± 3.1	26.94 ± 2.13	–
			Control (500 μl PBS was administered into the uterine cavity)	50	34.64 ± 3.0	28.53 ± 2.84	–
Salehpour 2020	Randomised controlled trial	patients who failed to conceive after 3 or more embryo transfers with high-quality embryos and candidates for frozenthawed embryo transfer (FET)	PRP (Intrauterine infusion of PRP was carried out 48 h before embryo transfer under ultrasound guidance)	49	35.73 ± 3.49	25.61 ± 3.13	1.9 ± 0.8
			Control (standard treatment)	48	34.95 ± 4.23	25.46 ± 2.68	1.7 ± 0.6
Zamaniyan 2021	Randomised controlled trial	women who unsuccessful to be pregnant after three or more high-quality embryo transfers undergoing frozen-thawed embryo transfer	PRP (Intrauterine infusion of platelet-rich plasma was performed 48 h before embryo transfer)	55	33.88 ± 6.32	26.49 ± 4.53	–
			Control (Another cycle was continued as described previously without platelet-rich plasma)	43	33.13 ± 5.00	25.03 ± 3.66	–

### CPR network meta-analysis

**Figure 4:**
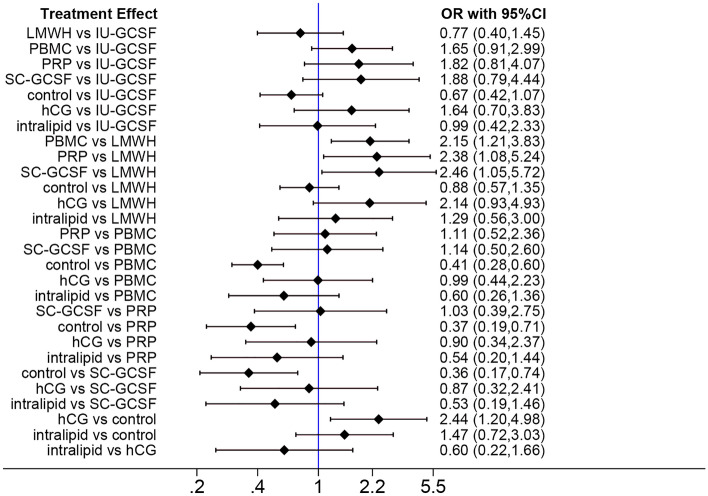
Network forest plot of CPR.

The results of the CPR network meta-analysis were indicated in Fig. 4. PBMC, PRP, SC-GCSF, and hCG administration could all significantly increase the CPR as compared with the control (PBMCs: OR 2.44; 95% CI 1.67–3.57; PRP: OR 2.70; 95% CI 1.41–5.26; SC-GCSF: OR 2.78; 95% CI 1.35–5.88; hCG: OR 2.44; 95% CI 1.20–4.98). Additionally, PBMCs, PRP, and SC-GCSF could also significantly increase the CPR as compared with LMWH (PBMCs: OR 2.15; 95% CI 1.21–3.83; PRP: OR 2.38; 95% CI 1.08–5.24; SC-GCSF: OR 2.46; 95% CI 1.05–5.72).

### LBR network meta-analysis

**Figure 5:**
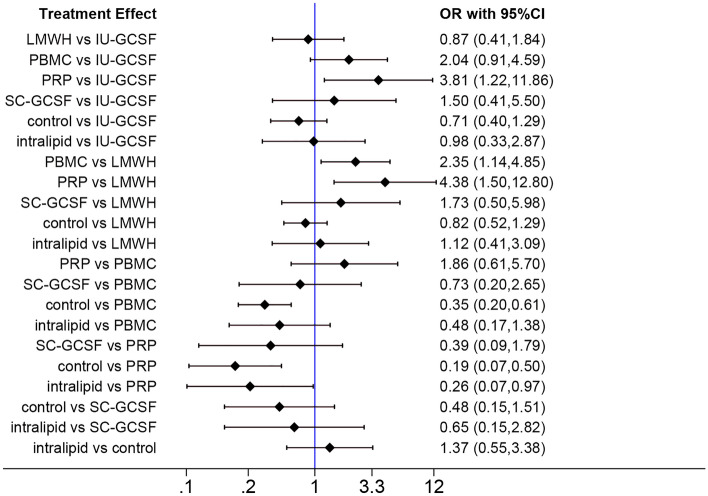
Network forest plot of LBR.

Nine of the included studies reported data on the LBR. The network meta-analysis outcomes implied that the administration of PBMCs and PRP led to a higher LBR in comparison with the control group (PBMCs: OR 2.86; 95% CI 1.64–5.00; PRP: OR 5.26; 95% CI 2.00–14.29) (Fig. 5). The effect of PRP on the LBR was significantly better than those of IU-GCSF (OR 3.81; 95% CI 1.22–11.86), LMWH (OR 4.38; 95% CI 1.50–12.90), or intralipid (OR 3.85; 95% CI 1.03–14.29), and the efficacy of PBMCs for improving the LBR was also significantly better than that of LMWH (OR 2.35; 95% CI 1.14–4.85).

### IR network meta-analysis

**Figure 6:**
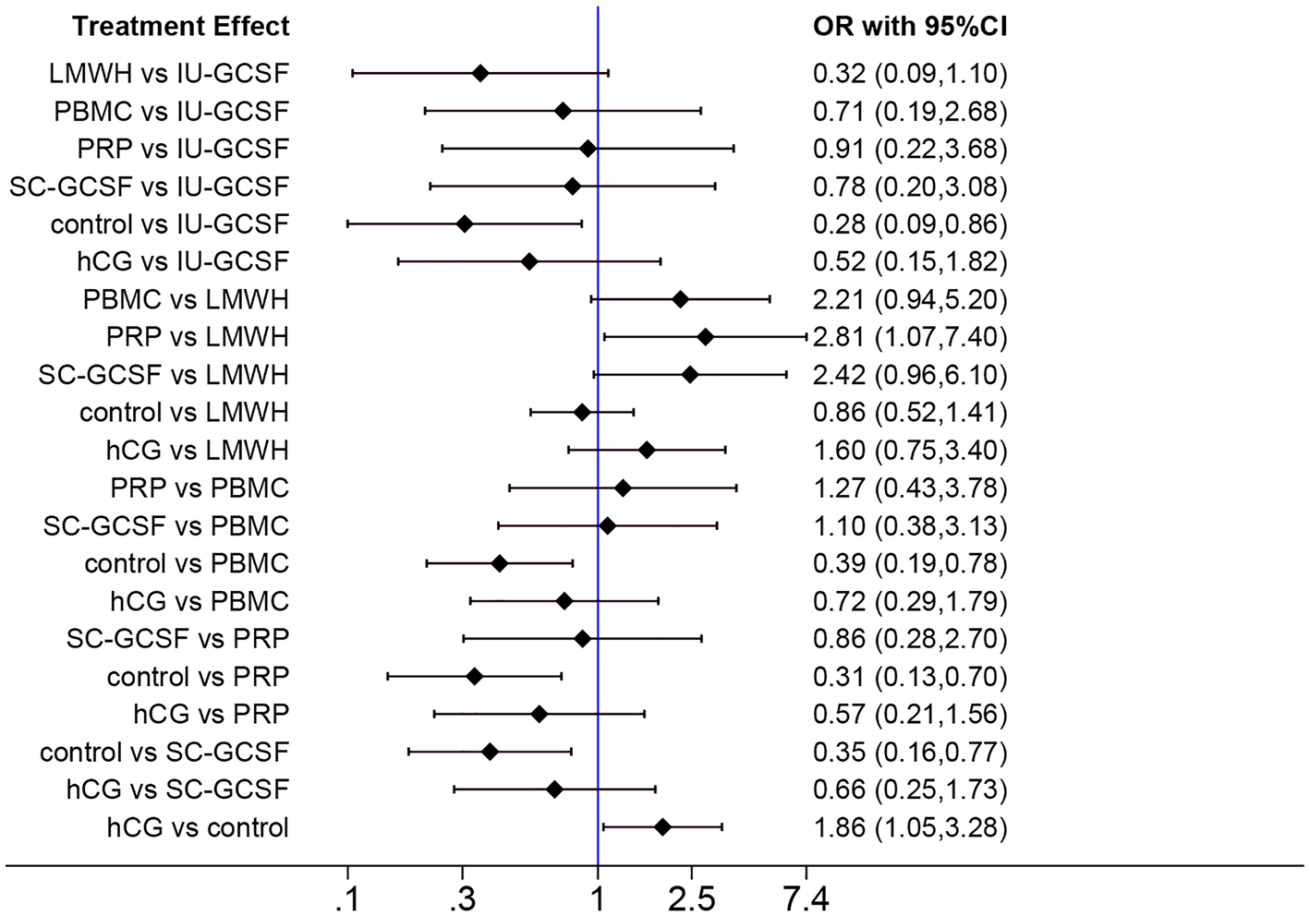
Network forest plot of IR.

We conducted a network meta-analysis on the nine studies that reported IR data. The results showed that IU-GCSF, PBMCs, PRP, SC-GCSF, and hCG were each significantly associated with a higher IR as compared with the control group (IU-GCSF: OR 3.57; 95% CI 1.16–11.1; PBMCs: OR 2.56; 95% CI 1.28–5.26; PRP: OR 3.23; 95% CI 1.43–7.69; SC-GCSF: OR 2.86; 95% CI 1.30–6.25; hCG: OR 1.86; 95% CI 1.05–3.28) (Fig. 6). Furthermore, PRP significantly improved the IR as compared with LMWH (OR 2.81; 95% CI 1.07–7.4).

**Figure 7:**
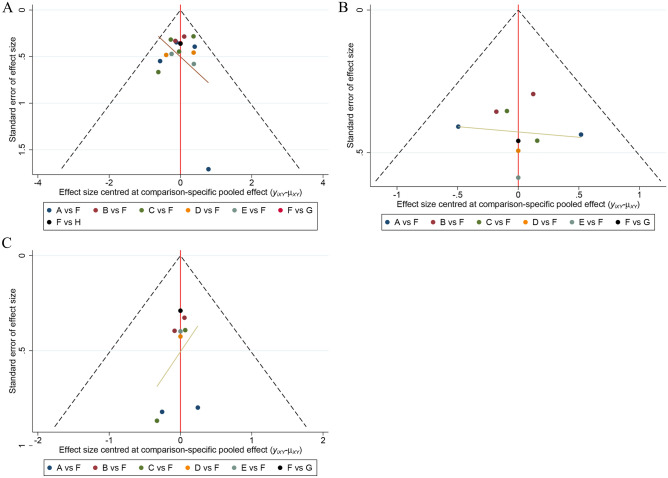
Funnel plot of CPR,LBR and IR.

The I^2^ values were 37.4% for the CPR, 16.1% for the LBR, and 50.8% for the IR (Figures S1–S3). Comparison-adjusted funnel plots of the network meta-analysis of each outcome suggested that there was no publication bias (Fig. 7). Furthermore, the node-splitting method was used for comparing the differences between direct and indirect evidence to assess inconsistency. No significant inconsistencies were found in the results (all *P* > 0.05), indicating that the results are reliable (details shown in Table S1).

### The ranking probability of SUCRA

The ranking probability of SUCRA for each treatment included in the network was shown in Table 2. In terms of the CPR, SC-GCSF was the most effective therapy (78.9%), while LMWH was the least effective therapy (18.3%). As far as the LBR is concerned, PRP was the most effective treatment (94.8%), and LMWH was the least effective (29.4%). Finally, regarding the IR, the most effective treatment was IU-GCSF (77.6%), and the least effective was LMWH (16.2%).

**Table 2:** SUCRA of CPR, LBR and IR.

Treatment	CPR	LBR	IR
IU-GCSF	37.1	39.5	77.6
LMWH	18.3	29.4	16.2
PBMC	73.1	78	63.4
PRP	77.9	94.8	75.9
SC-GCSF	78.9	60.4	68.1
Control	7	11	5.1
hCG	71.4		43.7
Intralipid	36.3	36.9	

## Discussion

The pregnancy rate has increased each year owing to the development of assisted reproductive technology, but there are still a number of patients who suffer from RIF^32–34^. Previous studies showed that uterine abnormalities; spermatic factor anomalies; genetic, hormonal, and metabolic pathologies; acquired thrombophilia; and autoimmune disorders are all possible causes of RIF^35^. However, RIF remains unexplained in approximately 30% of instances^36^. It has been reported that immune factors are crucial in the process of embryo implantation, and immunomodulatory therapies can improve the pregnancy outcomes of some patients with RIF^36^. Recently, there have been many studies conducted on the immune factors involved in the pathogenesis of RIF and immunotherapeutic methods, but there are differences in the efficacy and mechanisms of different preparations. Therefore, this study evaluated the efficacy of immunomodulatory therapies for improving the CPR and LBR of RIF patients through a network meta-analysis. Based on the outcomes of treated RIF patients, it was found that PBMCs and PRP are effective therapies for boosting the CPR and LBR. In comparison with the control group, treatment with PBMCs, PRP, SC-GCSF, or hCG significantly increased the CPR and IR, and PBMCs and PRP were significantly related with a higher LBR.

Previous research has demonstrated that RIF patients can benefit from immunomodulatory therapies, but there was still no direct or indirect comparison of the efficacy of different immunomodulatory therapies^15,37–40^. The present study evaluated the efficacy of five immunomodulatory therapies via a network meta-analysis system and found that SC-GCSF is the best therapy for improving the CPR, while IU-GCSF is the best option for improving the IR. Our results confirm the conclusions of Zhao et al. and Xie et al. Zhao et al. showed that the administration of G-CSF may have a favorable clinical effect on pregnancy outcomes. In addition, the best route by which to administer G-CSF may be a subcutaneous injection^41^. G-CSF, as a glycoprotein, belongs to the growth factor family. It was discovered to regulate the growth of the endometrium and to be involved in the occurrence of early endometriosis^42^. G-CSF has been shown to promote endometrial stem cells, mobilize bone marrow stem cells, and enhance endometrial development^43^. Xie et al. found that an intrauterine perfusion of G-CSF could significantly improve the IR as compared with control group^44^. However, there remains controversy regarding the ideal route of G-CSF administration, and the reasons for the different effects of these two administration routes have not yet been fully clarified. Therefore, more higher-quality studies are needed to clarify these phenomena.

In terms of the LBR, PRP has the best efficacy among the five assessed immunomodulatory therapies. We also discovered that PRP had a significantly better effect on the LBR than did IU-G-CSF, LMWH, and intralipid. Moreover, PRP can also increase the CPR and IR of RIF patients as compared with control group. PRP is composed of a high concentration of autologous platelets, normally 5–7 times greater than the platelet concentration in peripheral blood, which was collected by centrifuging peripheral whole blood^45^. PRP contains a variety of growth factors and cytokines, which may help regulate endometrial cell migration, attachment, proliferation, differentiation, and neovascularization, thereby having a beneficial effect on endometrial receptivity^46,47^. Amable et al. showed that, compared with whole blood plasma or platelet-poor plasma, the levels of 12 proteins (including six growth factors, three anti-inflammatory cytokines, and three pro-inflammatory cytokines) in activated PRP increased^48^. These cytokines and growth factors may boost the endometrium receptivity. Additionally, a mouse experiment showed that an autologous PRP intrauterine infusion accelerated and enhanced the regeneration of impaired endometrium and reduced endometrial fibrosis^49^. Owing to the limitation of the quantity of the studies, currently there is no meta-analysis to analyze the effect of PRP on the LBR, so additional high-standard studies are required to verify the benefits of PRP on the LBR.

An intrauterine infusion of PBMCs is also a good choice for RIF patients. PBMCs are mainly composed of T lymphocytes, B lymphocytes, and monocytes^50^. It has been reported that an infusion of PBMCs was able to regulate the production of a variety of cytokines and also promote the spread and invasion of blastocysts to the endometrium as well as the receptivity of the endometrium in vitro^39^. The results of a recent RCT indicate that PBMC infusion was an effective treatment strategy for RIF-related infertility^25^. Additionally, consistent with the results of our research, Maleki-Hajiagha et al. found that a PBMC infusion could increase the CPR and LBR of RIF patients^14^. Their study uncovered that PBMCs could significantly increase the CPR, LBR, and IR of RIF patients, as compared with the control group. The implantation promotion effect of PBMCs can be explained by a variety of mechanisms. It was reported that PBMCs can regulate the production of several cytokines, such as IL-1α, IL-1β, and TNF-α, and can promote the spread and invasion of blastocysts to the endometrium as well as the receptivity of the endometrium in vitro^51^. In addition, in vivo studies showed that the administration of PBMCs could promote implantation and clinical pregnancy rates and may optimize the in vitro fertilization results of patients with multiple failures from in vitro fertilization/ICSI^24,52^. Although our research indicates that its clinical effects were positive, adverse reactions should also be considered and will require further research for evaluation.

Our study has some limitations. First, no protocol was registered for this study. Second, conference abstracts and non-English language studies were excluded from this meta-analysis, and relatively few studies were included, with only one study on hCG. Therefore, there might be some potential local or other biases in the results. Third, the included studies may be biased, and undetermined hypercoagulative and immunological abnormalities were not investigated and intervened appropriately. Fourth, very few qualified studies reported the adverse events of their tested interventions, so it was lack of safety evaluation for the different drugs used in RIF treatment. Finally, there were differences in the dose of the same drug among different studies, but it was not feasible to further divide the studies into subgroups for analysis because of the restricted sample size.

## Conclusions

This network meta-analysis showed that PBMC, PRP, SC-GCSF, and hCG administration can each significantly increase the CPR and IR as compared to the control group. Furthermore, PBMC and PRP administration led to a higher LBR as compared with the control group. Our findings suggest that, among the different available immunotherapeutic medications for treating RIF, PBMC and PRP might provide the best therapeutic efficacy. Additional high-quality studies are necessary to verify the conclusions drawn from this research owing to its restricted number of included studies.

## Supplementary Information

Supplementary Figure S1.

Supplementary Figure S2.

Supplementary Figure S3.

Supplementary Information 1.

Supplementary Information 2.

Supplementary Information 3.

Supplementary Table S1.

## Data Availability

All data generated or analyzed during this study are included in this published article and its supplementary materials.
