# Supplementary figures and images for: The effectiveness of immunomodulatory therapies for patients with repeated implantation failure: a systematic review and network meta-analysis
Source: Sci Rep. 2022 Nov 1;12:18434. doi: 10.1038/s41598-022-21014-9 (PMC9626579; doi:10.1038/s41598-022-21014-9)

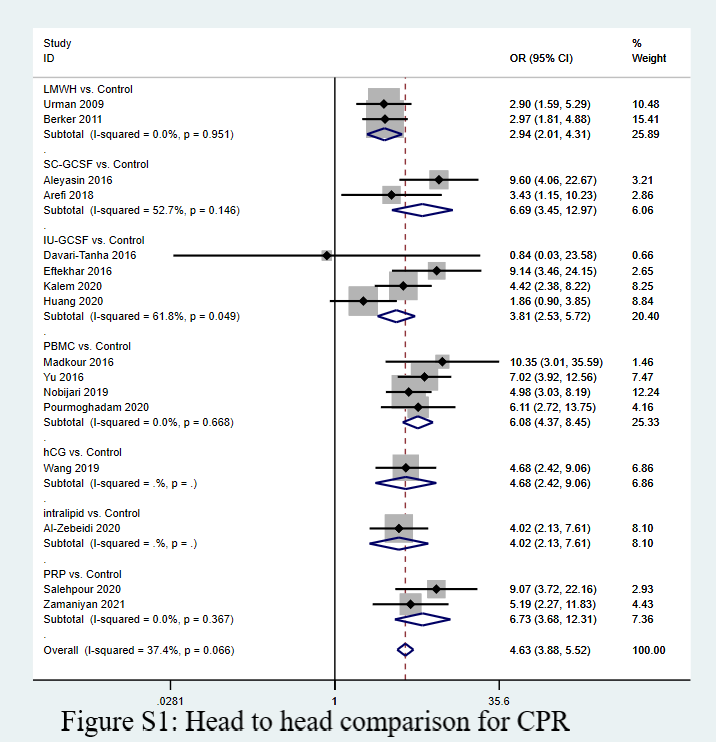

Supplement: Supplementary file 1 — Supplementary Figure S1. [file 41598_2022_21014_MOESM1_ESM.tif]

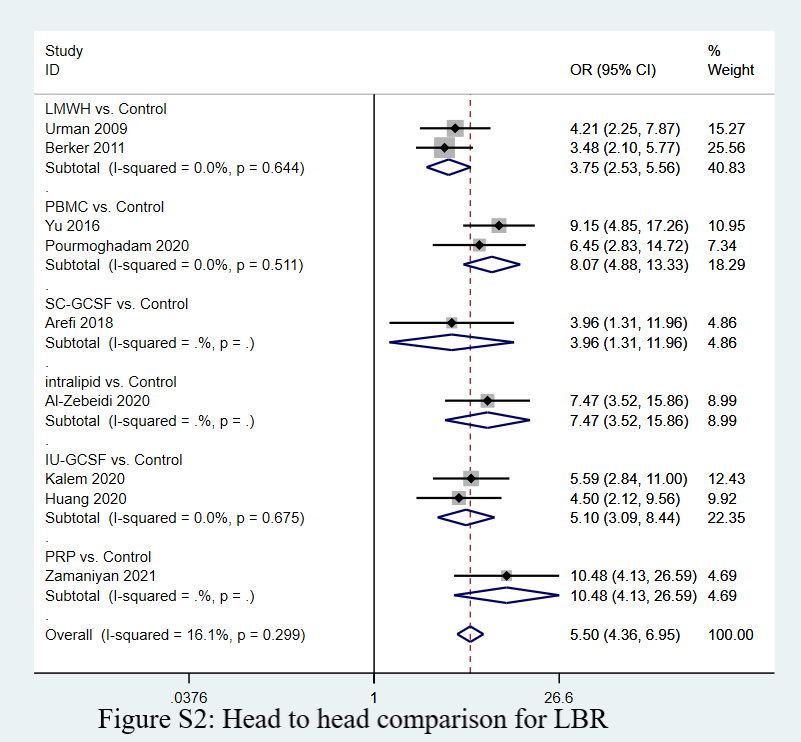

Supplement: Supplementary file 2 — Supplementary Figure S2. [file 41598_2022_21014_MOESM2_ESM.tif]

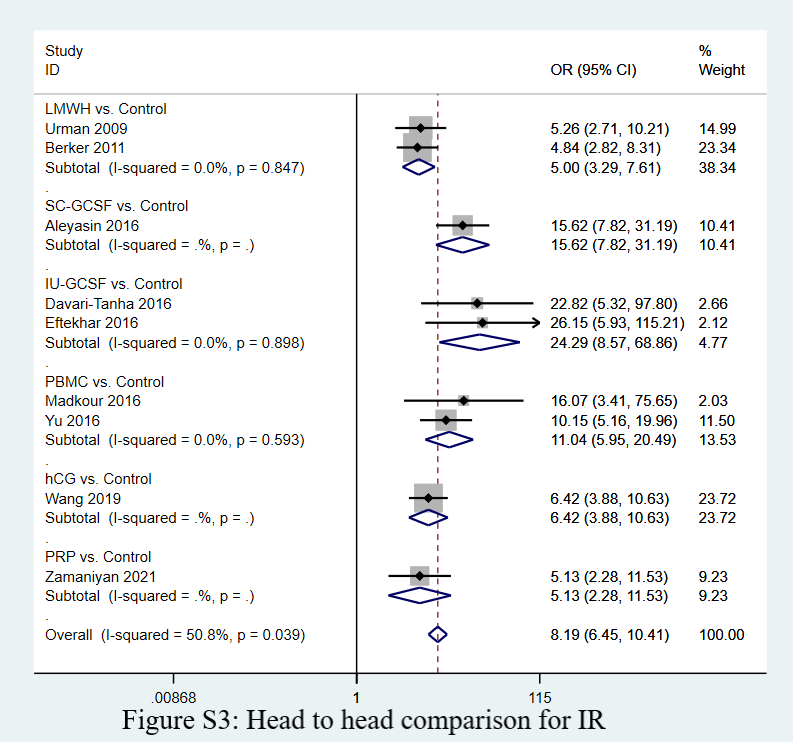

Supplement: Supplementary file 3 — Supplementary Figure S3. [file 41598_2022_21014_MOESM3_ESM.tif]
