# Supplementary material for: The effectiveness of immunomodulatory therapies for patients with repeated implantation failure: a systematic review and network meta-analysis
Source: Sci Rep. 2022 Nov 1;12:18434. doi: 10.1038/s41598-022-21014-9 (PMC9626579; doi:10.1038/s41598-022-21014-9)
Supplement: Supplementary file 5 — Supplementary Information 2. [file 41598_2022_21014_MOESM5_ESM.docx]

**Supplementary Material 1. Search strategy**

**Embase:**

#1 ‘implantation failure’:ab:ti

#2 ‘repeated implantation failure’:ab:ti

#3 ‘recurrent implantation failure’:ab:ti

#4 ‘RIF’:ab:ti

#5 #1 OR #2 OR #3 OR #4

#6 ‘intravenous immunoglobulin’:ab:ti

#7 ‘Antibodies, Intravenous’:ab:ti

#8 ‘Intravenous Antibodies’:ab:ti

#9 ‘Immune Globulin, Intravenous’:ab:ti

#10 ‘Intravenous Immune Globulin’:ab:ti

#11 ‘Intravenous Immunoglobulins’:ab:ti

#12 ‘Intravenous IG’:ab:ti

#13 ‘IV Immunoglobulins’:ab:ti

#14 ‘Immunoglobulins, IV’:ab:ti

#15 ‘IVIG’:ab:ti

#16 ‘IV Immunoglobulin’:ab:ti

#17 ‘Immunoglobulin, IV’:ab:ti

#18 ‘Intravenous Immunoglobulin’:ab:ti

#19 ‘Immunoglobulin, Intravenous’:ab:ti

#20 ‘Flebogamma DIF’:ab:ti

#21 ‘Gamunex’:ab:ti

#22 ‘Globulin N’:ab:ti

#23 ‘Intraglobin’:ab:ti

#24 ‘Intraglobin F’:ab:ti

#25 ‘Intravenous Immunoglobulins, Human’:ab:ti

#26 ‘Human Intravenous Immunoglobulins’:ab:ti

#27 ‘Immunoglobulins, Human Intravenous’:ab:ti

#28 ‘Immune Globulin Intravenous (Human)’:ab:ti

#29 ‘Immunoglobulins, Intravenous, Human’:ab:ti

#30 ‘Human Intravenous Immunoglobulin’:ab:ti

#31 ‘Immunoglobulin, Human Intravenous’:ab:ti

#32 ‘Intravenous Immunoglobulin, Human’:ab:ti

#33 ‘Gammagard’:ab:ti

#34 ‘Gamimune’:ab:ti

#35 ‘Gamimmune’:ab:ti

#36 ‘Modified Immune Globulin (Anti-Echovirus Antibody)’:ab:ti

#37 ‘Privigen’:ab:ti

#38 ‘Sandoglobulin’:ab:ti

#39 ‘Venoglobulin’:ab:ti

#40 ‘Venoglobulin-I’:ab:ti

#41 ‘Venoglobulin I’:ab:ti

#42 ‘Venimmune’:ab:ti

#43 ‘Iveegam’:ab:ti

#44 ‘Alphaglobin’:ab:ti

#45 ‘Endobulin’:ab:ti

#46 ‘Gamimune N’:ab:ti

#47 ‘Gamimmune N’:ab:ti

#48 ‘Gammonativ’:ab:ti

#49 ‘peripheral blood mononuclear cell’:ab:ti

#50 ‘PBMC’:ab:ti

#51 ‘Tacrolimus’:ab:ti

#52 ‘Prograf’:ab:ti

#53 ‘Prograft’:ab:ti

#54 ‘FR 900506’:ab:ti

#55 ‘FK 506’:ab:ti

#56 ‘granulocyte colony stimulating factor’:ab:ti

#58 ‘G-CSF’:ab:ti

#59 ‘autologous platelet-rich plasma’:ab:ti

#60 ‘autologous PRP’:ab:ti

#61 ‘intralipid’:ab:ti

#62 ‘Travamulsion’:ab:ti

#63 ‘Liposyn III’:ab:ti

#64 ‘Intrafat’:ab:ti

#65 ‘Intralipid’:ab:ti

#66 ‘human chorionic gonadotropin’:ab:ti

#67 ‘hCG’:ab:ti

#68 ‘low-molecular-weight heparin’:ab:ti

#69 ‘LMWH’:ab:ti

#70 ‘Heparin, Low Molecular Weight’:ab:ti

#71 ‘Aspirin’:ab:ti

#72 ‘Acetylsalicylic Acid’:ab:ti

#73 ‘Acylpyrin’:ab:ti

#74 ‘Aloxiprimum’:ab:ti

#75 ‘Colfarit’:ab:ti

#76 ‘Dispril’:ab:ti

#77 ‘Easprin’:ab:ti

#78 ‘Ecotrin’:ab:ti

#79 ‘Endosprin’:ab:ti

#80 ‘Magnecyl’:ab:ti

#81 ‘Micristin’:ab:ti

#82 ‘Polopirin’:ab:ti

#83 ‘Polopiryna’:ab:ti

#84 ‘Solprin’:ab:ti

#85 ‘Solupsan’:ab:ti

#86 ‘Zorprin’:ab:ti

#87 ‘Acetysal’:ab:ti

#88 ‘Glucocorticoids’:ab:ti

#89 #6 OR #7 OR #8 OR #9 OR #10 OR #11 OR #12 OR #13 OR #14 OR #15 OR #16 OR #17 OR #18 OR #19 OR #20 OR #21 OR #22 OR #23 OR #24 OR #25 OR #26 OR #27 OR #28 OR #29 OR #30 OR #31 OR #32 OR #33 OR #34 OR #35 OR #36 OR #37 OR #38 OR #39 OR #40 OR #41 OR #42 OR #43 OR #44 OR #45 OR #46 OR #47 OR #48 OR #49 OR #50 OR #51 OR #52 OR #53 OR #54 OR #55 OR #56 OR #57 OR #58 OR #59 OR #60 OR #61 OR #62 OR #63 OR #64 OR #65 OR #66 OR #67 OR #68 OR #69 OR #70 OR #71 OR #72 OR #73 OR #74 OR #75 OR #76 OR #77 OR #78 OR #79 OR #80 OR #81 OR #82 OR #83 OR #84 OR #85 OR #86 OR #87 OR #88

#90 #5 AND #89

**Web of Science:**

((implantation failure) or (repeated implantation failure) or (recurrent implantation failure) or (RIF)) and ((intravenous immunoglobulin) or (Antibodies, Intravenous) or (Intravenous Antibodies) or (Immune Globulin, Intravenous) or (Intravenous Immune Globulin) or (Intravenous Immunoglobulins) or (Intravenous IG) or (IV Immunoglobulins) or (Immunoglobulins, IV) or (IVIG) or (IV Immunoglobulin) or (Immunoglobulin, IV) or (Intravenous Immunoglobulin) or (Immunoglobulin, Intravenous) or (Flebogamma DIF) or (Gamunex) or (Globulin-N) or (Globulin N) or (Intraglobin) or (Intraglobin F) or (Intravenous Immunoglobulins, Human) or (Human Intravenous Immunoglobulins) or (Immunoglobulins, Human Intravenous) or (Immune Globulin Intravenous (Human)) or (Immunoglobulins, Intravenous, Human) or (Human Intravenous Immunoglobulin) or (Immunoglobulin, Human Intravenous) or (Intravenous Immunoglobulin, Human) or (Gammagard) or (Gamimune) or (Gamimmune) or (Modified Immune Globulin) or (Anti-Echovirus Antibody) or (Privigen) or (Sandoglobulin) or (Venoglobulin) or (Venoglobulin-I) or (Venoglobulin I) or (Venimmune) or (Iveegam) or (Alphaglobin) or (Endobulin) or (Gamimune N) or (Gamimmune N) or (Gammonativ) or (peripheral blood mononuclear cell) or (PBMC) or (Tacrolimus) or (Prograf) or (Prograft) or (FR 900506) or (FK 506) or (granulocyte colony stimulating factor) or (G-CSF) or (autologous platelet-rich plasma ) or (autologous PRP) or (intralipid) or (Travamulsion) or (Liposyn III) or (Intrafat) or (Intralipid) or (human chorionic gonadotropin) or (hCG) or (low-molecular-weight heparin) or (LMWH) or (Heparin, Low Molecular Weight) or (Aspirin) or (Acetylsalicylic Acid) or (Acylpyrin) or (Aloxiprimum) or (Colfarit) or (Dispril) or (Easprin) or (Ecotrin) or (Endosprin) or (Magnecyl) or (Micristin) or (Polopirin) or (Polopiryna) or (Solprin) or (Solupsan) or (Zorprin) or (Acetysal) or (Glucocorticoids))

**Pubmed:**

((((((((((((((((((((((((((((((((((((((((((((((((((((((((((((((((((((((((((((((((intravenous immunoglobulin[Title/Abstract]) OR (Antibodies, Intravenous[Title/Abstract])) OR (Intravenous Antibodies[Title/Abstract])) OR (Immune Globulin, Intravenous[Title/Abstract])) OR (Intravenous Immune Globulin[Title/Abstract])) OR (Intravenous Immunoglobulins[Title/Abstract])) OR (Intravenous IG[Title/Abstract])) OR (IV Immunoglobulins[Title/Abstract])) OR (Immunoglobulins, IV[Title/Abstract])) OR (IVIG[Title/Abstract])) OR (IV Immunoglobulin[Title/Abstract])) OR (Immunoglobulin, IV[Title/Abstract])) OR (Intravenous Immunoglobulin[Title/Abstract])) OR (Immunoglobulin, Intravenous[Title/Abstract])) OR (Flebogamma DIF[Title/Abstract])) OR (Gamunex[Title/Abstract])) OR (Globulin-N[Title/Abstract])) OR (Globulin N[Title/Abstract])) OR (Intraglobin[Title/Abstract])) OR (Intraglobin F[Title/Abstract])) OR (Intravenous Immunoglobulins, Human[Title/Abstract])) OR (Human Intravenous Immunoglobulins[Title/Abstract])) OR (Immunoglobulins, Human Intravenous[Title/Abstract])) OR (Immune Globulin Intravenous (Human)[Title/Abstract])) OR (Immunoglobulins, Intravenous, Human[Title/Abstract])) OR (Human Intravenous Immunoglobulin[Title/Abstract])) OR (Immunoglobulin, Human Intravenous[Title/Abstract])) OR (Intravenous Immunoglobulin, Human[Title/Abstract])) OR (Gammagard[Title/Abstract])) OR (Gamimune[Title/Abstract])) OR (Gamimmune[Title/Abstract])) OR (Privigen[Title/Abstract])) OR (Sandoglobulin[Title/Abstract])) OR (Venoglobulin[Title/Abstract])) OR (Venoglobulin-I[Title/Abstract])) OR (Venimmune[Title/Abstract])) OR (Iveegam[Title/Abstract])) OR (Alphaglobin[Title/Abstract])) OR (Endobulin[Title/Abstract])) OR (Gamimune N[Title/Abstract])) OR (Gammonativ[Title/Abstract])) OR (peripheral blood mononuclear cell[Title/Abstract])) OR (PBMC[Title/Abstract])) OR (Tacrolimus[Title/Abstract])) OR (Prograf[Title/Abstract])) OR (Prograf[Title/Abstract])) OR (FR 900506[Title/Abstract])) OR (FK 506[Title/Abstract])) OR (granulocyte colony stimulating factor[Title/Abstract])) OR (G-CSF[Title/Abstract])) OR (autologous platelet-rich plasma[Title/Abstract])) OR (autologous PRP[Title/Abstract])) OR (intralipid[Title/Abstract])) OR (Travamulsion[Title/Abstract])) OR (Liposyn III[Title/Abstract])) OR (Intrafat[Title/Abstract])) OR (Intralipid[Title/Abstract])) OR (human chorionic gonadotropin[Title/Abstract])) OR (hCG[Title/Abstract])) OR (low-molecular-weight heparin[Title/Abstract])) OR (LMWH[Title/Abstract])) OR (Heparin, Low Molecular Weight[Title/Abstract])) OR (Aspirin[Title/Abstract])) OR (Acetylsalicylic Acid[Title/Abstract])) OR (Acylpyrin[Title/Abstract])) OR (Aloxiprimum[Title/Abstract])) OR (Colfarit[Title/Abstract])) OR (Dispril[Title/Abstract])) OR (Easprin[Title/Abstract])) OR (Ecotrin[Title/Abstract])) OR (Endosprin[Title/Abstract])) OR (Magnecyl[Title/Abstract])) OR (Micristin[Title/Abstract])) OR (Polopirin[Title/Abstract])) OR (Polopiryna[Title/Abstract])) OR (Solprin[Title/Abstract])) OR (Solupsan[Title/Abstract])) OR (Zorprin[Title/Abstract])) OR (Acetysal[Title/Abstract])) OR (Glucocorticoids[Title/Abstract])) AND ((((implantation failure[Title/Abstract]) OR (repeated implantation failure[Title/Abstract])) OR (recurrent implantation failure[Title/Abstract])) OR (RIF[Title/Abstract]))
