# Supplementary material for: The effectiveness of immunomodulatory therapies for patients with repeated implantation failure: a systematic review and network meta-analysis
Source: Sci Rep. 2022 Nov 1;12:18434. doi: 10.1038/s41598-022-21014-9 (PMC9626579; doi:10.1038/s41598-022-21014-9)
Supplement: Supplementary file 6 — Supplementary Information 3. [file 41598_2022_21014_MOESM6_ESM.docx]

**Supplementary Material 3. Full text reading exclusion reasons**

**Non-RIF study (15):**

1. Moraru M, Carbone J, Alecsandru D, Castillo-Rama M, García-Segovia A, Gil J, et al. Intravenous immunoglobulin treatment increased live birth rate in a Spanish cohort of women with recurrent reproductive failure and expanded CD56(+) cells. Am J Reprod Immunol. 2012;68(1):75-84. Epub 2012/04/19. doi: 10.1111/j.1600-0897.2012.01135.x. PubMed PMID: 22509929.

2. Ramos-Medina R, García-Segovia A, Gil J, Carbone J, Aguarón de la Cruz A, Seyfferth A, et al. Experience in IVIg therapy for selected women with recurrent reproductive failure and NK cell expansion. Am J Reprod Immunol. 2014;71(5):458-66. Epub 2014/03/13. doi: 10.1111/aji.12217. PubMed PMID: 24612159.

3. Ramos-Medina R, García-Segovia A, Gil J, Carbone J, Aguarón de la Cruz A, Seyfferth A, et al. Experience in IVIg therapy for selected women with recurrent reproductive failure and NK cell expansion. American Journal of Reproductive Immunology. 2014;71(5):458-66. doi: 10.1111/aji.12217. PubMed Central PMCID: PMCCSL Behring(Switzerland)

Grifols(Spain).

4. Russell P, Anderson L, Lieberman D, Tremellen K, Yilmaz H, Cheerala B, et al. The distribution of immune cells and macrophages in the endometrium of women with recurrent reproductive failure I: Techniques. Journal of Reproductive Immunology. 2011;91(1-2):90-102. doi: 10.1016/j.jri.2011.03.013. PubMed PMID: WOS:000295496100013.

5. Nyborg KM, Kolte AM, Larsen EC, Christiansen OB. Immunomodulatory treatment with intravenous immunoglobulin and prednisone in patients with recurrent miscarriage and implantation failure after in vitro fertilization/intracytoplasmic sperm injection. Fertil Steril. 2014;102(6):1650-5.e1. Epub 2014/09/27. doi: 10.1016/j.fertnstert.2014.08.029. PubMed PMID: 25256927.

6. Scarpellini F, Sbracia M. Use of granulocyte colony-stimulating factor for the treatment of unexplained recurrent miscarriage: a randomised controlled trial. Hum Reprod. 2009;24(11):2703-8. Epub 2009/07/21. doi: 10.1093/humrep/dep240. PubMed PMID: 19617208.

7. Scarpellini F, Sbracia M, Balili A. The role of G-CSF treatment in recurrent miscarriage on the expression of FOXP3, VEGF, VEGF-R2 and c-kit in first trimester pregnancy specimens. Reproductive Sciences. 2017;24(1):161A. doi: 10.1177/1933719117699773.

8. Woon EV, Day A, Bracewell-Milnes T, Male V, Johnson M. Immunotherapy to improve pregnancy outcome in women with abnormal natural killer cell levels/activity and recurrent miscarriage or implantation failure: A systematic review and meta-analysis. Journal of Reproductive Immunology. 2020;142. doi: 10.1016/j.jri.2020.103189.

9. Zafardoust S, Akhondi MM, Sadeghi MR, Mohammadzadeh A, Karimi A, Jouhari S, et al. Efficacy of intrauterine injection of granulocyte colony stimulating factor (G-CSF) on treatment of unexplained recurrent miscarriage. Human Reproduction. 2016;31:i171. doi: 10.1093/humrep/31.Supplement_1.1.

10. Singh N, Davis AA, Kumar S, Kriplani A. The effect of administration of intravenous intralipid on pregnancy outcomes in women with implantation failure after IVF/ICSI with non-donor oocytes: A randomised controlled trial. Eur J Obstet Gynecol Reprod Biol. 2019;240:45-51. Epub 2019/06/23. doi: 10.1016/j.ejogrb.2019.06.007. PubMed PMID: 31228675.

11. Singh N, Davis A, Kumar S. Effect of intravenous intralipid on implantation rate in women with previous implantation failure after IVF/ICSI: a randomised controlled trial. Bjog-an International Journal of Obstetrics and Gynaecology. 2019;126:203-. PubMed PMID: WOS:000471271000407.

12. Nakagawa K. Immunosuppressive treatment with tacrolimus improves reproductive outcome of women with repeated implantation failures (RIF) who have elevated Th1/Th2 cell ratios. American Journal of Reproductive Immunology. 2019;81:88. doi: 10.1111/aji.11_13122.

13. Singh N, Davis A, Venamail P. Intravenous intralipid on implantation rate in women with implantation failure after IVF/ICSI. International Journal of Gynecology and Obstetrics. 2018;143:347. doi: 10.1002/ijgo.12582.

14. Singh N, Davis A, Kriplani A, Kumar S. Effect of administration of intravenous intralipid on implantation rate in women with implantation failure after IVF/ICSI: A randomized controlled trial. Human Reproduction. 2018;33:i82-i3. doi: 10.1093/humrep/33.Supplement_1.1.

15. Zamaniyan M, Peyvandi S, Heidaryan Gorji H, Moradi S, Jamal J, Yahya Poor Aghmashhadi F, et al. Effect of platelet-rich plasma on pregnancy outcomes in infertile women with recurrent implantation failure: a randomized controlled trial. Gynecol Endocrinol. 2021;37(2):141-5. Epub 2020/05/05. doi: 10.1080/09513590.2020.1756247. PubMed PMID: 32363968.

**Not RCT (6):**

1. Ahmadi M, Abdolmohammadi-Vahid S, Ghaebi M, Aghebati-Maleki L, Dolati S, Farzadi L, et al. Regulatory T cells improve pregnancy rate in RIF patients after additional IVIG treatment. Systems Biology in Reproductive Medicine. 2017;63(6):350-9. doi: 10.1080/19396368.2017.1390007.

2. Chang Y, Li J, Wei LN, Pang J, Chen J, Liang X. Autologous platelet-rich plasma infusion improves clinical pregnancy rate in frozen embryo transfer cycles for women with thin endometrium. Medicine (Baltimore). 2019;98(3):e14062. Epub 2019/01/18. doi: 10.1097/md.0000000000014062. PubMed PMID: 30653117; PubMed Central PMCID: PMCPMC6370111 the submitted work.

3. Ho YK, Chen HH, Huang CC, Lee CI, Lin PY, Lee MS, et al. Peripheral CD56+CD16+ NK Cell Populations in the Early Follicular Phase Are Associated With Successful Clinical Outcomes of Intravenous Immunoglobulin Treatment in Women With Repeated Implantation Failure. Frontiers in Endocrinology. 2020;10. doi: 10.3389/fendo.2019.00937.

4. Liu X, Ma D, Wang W, Qu Q, Zhang N, Wang X, et al. Intrauterine administration of human chorionic gonadotropin improves the live birth rates of patients with repeated implantation failure in frozen-thawed blastocyst transfer cycles by increasing the percentage of peripheral regulatory T cells. Archives of Gynecology and Obstetrics. 2019;299(4):1165-72. doi: 10.1007/s00404-019-05047-6.

5. Lodigiani C, Di Micco P, Ferrazzi P, Librè L, Arfuso V, Polatti F, et al. Low-molecular-weight heparin in women with repeated implantation failure. Women's Health. 2011;7(4):425-31. doi: 10.2217/whe.11.38.

6. Mehrafza M, Kabodmehri R, Nikpouri Z, Pourseify G, Raoufi A, Eftekhari A, et al. Comparing the Impact of Autologous Platelet-rich Plasma and Granulocyte Colony Stimulating Factor on Pregnancy Outcome in Patients with Repeated Implantation Failure. J Reprod Infertil. 2019;20(1):35-41. Epub 2019/03/13. PubMed PMID: 30859080; PubMed Central PMCID: PMCPMC6386791.

**Conference proceedings (20)**

1. Abdolmohammadi-Vahid S, Ghaebi M, Ahmadi M, Abdollahifard S, Danaii S, Mousapour P, et al. Intravenous immunoglobulin (IVIG) modulates regulatory T cells and improves pregnancy outcome in patients with repeated implantation failure (RIF). International Journal of Reproductive BioMedicine. 2017;15(4):75-6.

2. Abedi Asl Z. The efficacy of systemic administration of granulocyte colony stimulating factor (GCSF) on the in vitro fertilization (IVF) success in women with repeated implantation failure. Fertility and Sterility. 2015;104(3):e61.

3. Akhondi MM, Sadeghi MR, Mohammadzadeh F, Karimi A, Jouhari S, Ansaripour S, et al. Efficacy of intrauterine injection of granulocyte colony stimulating factor (GCSF) on treatment of unexplained recurrent miscarriage. Journal of Reproduction and Infertility. 2016;17(2):48-9.

4. Ansaripour S, Karimi A, Mokhtar S, Sadeghi MR, Kamali K. A randomized clinical trial oof intrauterine gcs in unexplained repeated implantation failure (RIF) patients: May intrauterine gcsf improve clinical & ongoing pregnancy rate or decrease abortion? Journal of Reproduction and Infertility. 2018;19(2):54.

5. Arefi S, Fazeli E, Esfahani M, Borhani N, Yamini N, Hosseini A, et al. GCSF may improve pregnancy outcome in blastocyst embryo transfer patients with history of unexplained implantation failure and normal endometrium. A randomized control trial. Journal of Reproduction and Infertility. 2018;19(2):138-9.

6. Obidniak D, Gzgzyan A, Dzhemlikhanova L, Feoktistov A. Effect of colony-stimulating growth factor on outcome of frozen-thawed embryo transfer in patients with repeated implantation failure. Fertility and Sterility. 2016;106:e134-e5.

7. Gamaleldin I, Gomaa MF, Shafik A, Akande V. Intralipid infusion does not improve live birth rates in women with unexplained recurrent implantation failure and may increase the risk of congenital malformations, a double-blinded randomised controlled trial. BJOG: An International Journal of Obstetrics and Gynaecology. 2018;125:31-2. doi: 10.1111/1471-0528.15493.

8. Ghaebi M, Abdolmohammadi-Vahid S, Ahmadi M, Nikmaram A, Farzadi L, Ghasemzadeh A, et al. The efficacy of intravenous immunoglobulin (IVIG) in pregnancy success and modulation of Th17 responses in women with recurrent implantation failure (RIF). International Journal of Reproductive BioMedicine. 2017;15(4):77.

9. Hamdi K, Danaii S, Ghasemzadeh A, Farzadi L, Abdollahi S, Nouri M. The role of heparin on embryo implantation rate in recurrent implantation failure cases without history of thrombophilia. Iranian Journal of Reproductive Medicine. 2014;12(6):6.

10. Holschbach V, Kuon RJ, Goeggl T, Daniel V, Weber M, Markert UR, et al. Intravenous lipid administration in RPL and RIF patients with elevated uterine natural killer cells. Human Reproduction. 2014;29:i140. doi: 10.1093/humrep/29.Supplement_1.1.

11. Hosseinisadat R. Effect of granulocyte colony stimulating factor (G-CSF) on IVF outcomes in normal infertile women. Journal of Reproduction and Infertility. 2016;17(2):206.

12. Jubiz G, Salazar M, Sung N, Yu M, Skariah A, Jubiz G, et al. Reproductive outcomes of women with recurrent pregnancy loss and repeated implantation failure (RIF) are significantly improved with immune modulatory treatment. American Journal of Reproductive Immunology. 2019;81:70. doi: 10.1111/aji.69_13120.

13. Kim CH, You RM, Nah HY, Kang HJ, Kim S, Chae HD, et al. Effect of granulocyte colony-stimulating factor on pregnancy outcome following IVF/ICSI in patients with repeated implantation failure. Human Reproduction. 2011;26:i244. doi: 10.1093/humrep/26.s1.82.

14. Knieper C, Franz C, Hirv K, Meri O, Santjohanser C, Würfel W, et al. G-CSF as new treatment option in patients with recurrent implantation failure. Journal of Reproductive Immunology. 2011;90(2):180. doi: 10.1016/j.jri.2011.06.091.

15. Konar H, Chakraborty B, Wasim S. Intrauterine infusion of autologous PBMC and pregnancy outcome in patients with repeated ivf failure. International Journal of Gynecology and Obstetrics. 2018;143:347. doi: 10.1002/ijgo.12582.

16. Kong N, Song T, Liu J. Effects of serum progesterone and LH levels before HCG triggering on clinical pregnancy outcomes of modified natural frozen-thawed embryo transfer cycles. Fertility and Sterility. 2019;112(3):e198-e9. doi: 10.1016/j.fertnstert.2019.07.636.

17. Koo HS, Min EG, Cha SH, Yi HJ, Koong MK, Yang KM. Efficacy of low molecular weighted heparin (LMWH) treatment in repeated implantation failure (RIF) patients with decreased uterine blood flow. Fertility and Sterility. 2014;102(3):e233. doi: 10.1016/j.fertnstert.2014.07.792.

18. Lodigiani C, Ferrazzi P, Libré L, Banfi E, Cafaro L, Morenghi M, et al. Low-molecular-weight heparin in pregnancies after art: A randomized study. Journal of Thrombosis and Haemostasis. 2015;13:681-2. doi: 10.1111/jth.12993.

19. Miraj S, Eftekhar M, Farid Mojtahedi M. Efficacy of transvaginal perfusion of granulocyte colony stimulating factor on recurrent implantation failure: Randomized control trial. Iranian Journal of Reproductive Medicine. 2015;13(4):16.

20. Neghab N, Eftekhar M, Naghshineh E, Khani P. Pregnancy outcomes in women with history of repeated implantation failure after intrauterine infusion of autologous platelet-rich plasma (PRP) in frozen-thawed cycles. International Journal of Reproductive BioMedicine. 2017;15(4):22.

**Others (13):**

1. Okitsu O, Kiyokawa M, Oda T, Miyake K, Sato Y, Fujiwara H. Intrauterine administration of autologous peripheral blood mononuclear cells increases clinical pregnancy rates in frozen/thawed embryo transfer cycles of patients with repeated implantation failure. Journal of Reproductive Immunology. 2011;92(1-2):82-7. doi: 10.1016/j.jri.2011.07.001.

2. Asl ZA. The efficacy of systemic administration of granulocyte colony stimulating factor (GCSF) on the in vitro fertilization (IVF) success in women with repeated implantation failure. Fertil Steril. 2015;104:e61.

3. Li S, Wang J, Cheng Y, Zhou D, Yin T, Xu W, et al. Intrauterine administration of hCG-activated autologous human peripheral blood mononuclear cells (PBMC) promotes live birth rates in frozen/thawed embryo transfer cycles of patients with repeated implantation failure. Journal of Reproductive Immunology. 2017;119:15-22. doi: 10.1016/j.jri.2016.11.006.

4. Eftekhar M MS, Mojtahedi MF, Neghab N. Effect of colony-stimulating growth factor on outcome of frozen-thawed embryo transfer in patients with repeated implantation failure. Fertil Steril. 2016;106(3).

5. Yoshioka S, Fujiwara H, Nakayama T, Kosaka K, Mori T, Fujii S. Intrauterine administration of autologous peripheral blood mononuclear cells promotes implantation rates in patients with repeated failure of IVF-embryo transfer. Human Reproduction. 2006;21(12):3290-4. doi: 10.1093/humrep/del312. PubMed PMID: WOS:000242271600038.

6. Ho YK, Chen HH, Huang CC, Lee CI, Lin PY, Lee MS, et al. Peripheral CD56(+)CD16(+) NK Cell Populations in the Early Follicular Phase Are Associated With Successful Clinical Outcomes of Intravenous Immunoglobulin Treatment in Women With Repeated Implantation Failure. Front Endocrinol (Lausanne). 2019;10:937. Epub 2020/02/11. doi: 10.3389/fendo.2019.00937. PubMed PMID: 32038492; PubMed Central PMCID: PMCPMC6985091.

7. Dieamant F, Vagnini LD, Petersen CG, Mauri AL, Renzi A, Petersen B, et al. New therapeutic protocol for improvement of endometrial receptivity (PRIMER) for patients with recurrent implantation failure (RIF) - A pilot study. Jornal Brasileiro de Reproducao Assistida. 2019;23(3):250-4. doi: 10.5935/1518-0557.20190035. PubMed Central PMCID: PMCBesins Healthcare(Brazil)

Biosintetica.

8. Coksuer H, Akdemir Y, Ulas Barut M. Improved in vitro fertilization success and pregnancy outcome with autologous platelet-rich plasma treatment in unexplained infertility patients that had repeated implantation failure history. Gynecological Endocrinology. 2019;35(9):815-8. doi: 10.1080/09513590.2019.1597344. PubMed Central PMCID: PMCSerono(Switzerland)

Merck Serono(Germany).

9. Martini AE, Jasulaitis S, Fogg LF, Uhler ML, Hirshfeld-Cytron JE. Evaluating the Utility of Intralipid Infusion to Improve Live Birth Rates in Patients with Recurrent Pregnancy Loss or Recurrent Implantation Failure. Journal of human reproductive sciences. 2018;11(3):261-8. doi: 10.4103/jhrs.JHRS_28_18. PubMed PMID: MEDLINE:30568356.

10. Huang P, Wei L, Li X, Qin A. Effects of intrauterine perfusion of human chorionic gonadotropin in women with different implantation failure numbers. American Journal of Reproductive Immunology. 2018;79(2). doi: 10.1111/aji.12809.

11. Ghasemnejad-berenji H, Novin MG, Hajshafiha M, Nazarian H, Hashemi SM, Ilkhanizadeh B, et al. Immunomodulatory effects of hydroxychloroquine on Th1/Th2 balance in women with repeated implantation failure. Biomedicine & Pharmacotherapy. 2018;107:1277-85. doi: 10.1016/j.biopha.2018.08.027. PubMed PMID: WOS:000445036200140.

12. Al-Zebeidi J, Lary S, Al-Jaroudi D. The effect of fat emulsion intralipid 20% in reproductive outcome for patients with multiple implantation failure. International Journal of Women's Health and Reproduction Sciences. 2018;6(2):144-9. doi: 10.15296/ijwhr.2018.24.

13. Rahman A, Francomano D, Sagnella F, Lisi F, Manna C. The effect on clinical results of adding recombinant LH in late phase of ovarian stimulation of patients with repeated implantation failure: A pilot study. European Review for Medical and Pharmacological Sciences. 2017;21(23):5485-90.
