# Supplementary material for: The effectiveness of immunomodulatory therapies for patients with repeated implantation failure: a systematic review and network meta-analysis
Source: Sci Rep. 2022 Nov 1;12:18434. doi: 10.1038/s41598-022-21014-9 (PMC9626579; doi:10.1038/s41598-022-21014-9)
Supplement: Supplementary file 7 — Supplementary Table S1. [file 41598_2022_21014_MOESM7_ESM.docx]

Table S1. Consistency Check Results

| Side | Direct | | Indirect | | Difference | | tau | |
| --- | --- | --- | --- | --- | --- | --- | --- | --- |
|  | Coef. | Std. Err. | Coef. | Std. Err. | Coef. | Std. Err. | P>\|z\| |  |
| **CPR** |  |  |  |  |  |  |  |  |
| IU-GCSF VS. control | -0.40039 | 0.236918 | -0.41669 | 19.42819 | 0.016291 | 19.42962 | 0.999 | 0.07247 |
| LMWH VS. control | -0.13267 | 0.222542 | -0.7996 | 92.36828 | 0.666934 | 92.36854 | 0.994 | 0.072484 |
| PBMC VS. control | -0.89933 | 0.194673 | -0.75754 | 68.54593 | -0.14179 | 68.54625 | 0.998 | 0.072451 |
| PRP VS. control | -0.99934 | 0.3357 | -0.65225 | 96.9084 | -0.3471 | 96.90927 | 0.997 | 0.072439 |
| SC-GCSF VS. control | -1.03112 | 0.369511 | -0.6096 | 95.1713 | -0.42152 | 95.17242 | 0.996 | 0.072448 |
| control VS. hCG | 0.892637 | 0.363627 | 0.760135 | 128.3065 | 0.132501 | 128.3071 | 0.999 | 0.072446 |
| control VS. intralipid | 0.387527 | 0.368165 | 0.787548 | 134.879 | -0.40002 | 134.8795 | 0.998 | 0.072483 |
| **LBR** |  |  |  |  |  |  |  |  |
| IU-GCSF VS. control | -0.33754 | 0.301417 | -0.45204 | 26.49953 | 0.114504 | 26.50147 | 0.997 | 0.048025 |
| LMWH VS. control | -0.19777 | 0.232355 | -0.67156 | 97.27681 | 0.473797 | 97.27608 | 0.996 | 0.047897 |
| PBMC VS. control | -1.05273 | 0.283461 | -0.57549 | 101.6708 | -0.47724 | 101.6716 | 0.996 | 0.047851 |
| PRP VS. control | -1.67398 | 0.495669 | -0.25001 | 131.9915 | -1.42397 | 131.9932 | 0.991 | 0.047974 |
| SC-GCSF VS. control | -0.7442 | 0.589542 | -0.48926 | 126.8904 | -0.25495 | 126.8925 | 0.998 | 0.047845 |
| control VS. intralipid | 0.312795 | 0.461359 | 0.658594 | 171.7827 | -0.3458 | 171.7834 | 0.998 | 0.0479 |
| **IR** |  |  |  |  |  |  |  |  |
| IU-GCSF VS. control | -1.27921 | 0.573012 | -0.4007 | 29.48955 | -0.87851 | 29.49512 | 0.976 | 3.03E-08 |
| LMWH VS. control | -0.15131 | 0.251979 | -2.55517 | 108.0604 | 2.403861 | 108.0607 | 0.982 | 2.82E-09 |
| PBMC VS. control | -0.94322 | 0.357254 | -2.44677 | 121.996 | 1.50355 | 121.9969 | 0.99 | 4.90E-08 |
| PRP VS. control | -1.18377 | 0.425385 | -2.41167 | 126.5219 | 1.227897 | 126.5228 | 0.992 | 1.38E-08 |
| SC-GCSF VS. control | -1.03615 | 0.398313 | -2.37846 | 191.1213 | 1.342311 | 191.122 | 0.994 | 3.06E-09 |
| control VS. hCG | 0.621235 | 0.289786 | 2.508965 | 149.191 | -1.88773 | 149.1914 | 0.99 | 5.91E-09 |
